# Cross-inhibition of pathogenic agents and the host proteins they exploit

**DOI:** 10.1038/srep34846

**Published:** 2016-10-05

**Authors:** Leeor Zilbermintz, William Leonardi, Sharon H. Tran, Josue Zozaya, Alyssa Mathew-Joseph, Spencer Liem, Anastasia Levitin, Mikhail Martchenko

**Affiliations:** 1 Keck Graduate Institute, Claremont, CA 91711, USA.

## Abstract

The major limitations of pathogen-directed therapies are the emergence of drug-resistance and their narrow spectrum of coverage. A recently applied approach directs therapies against host proteins exploited by pathogens in order to circumvent these limitations. However, host-oriented drugs leave the pathogens unaffected and may result in continued pathogen dissemination. In this study we aimed to discover drugs that could simultaneously cross-inhibit pathogenic agents, as well as the host proteins that mediate their lethality. We observed that many pathogenic and host-assisting proteins belong to the same functional class. In doing so we targeted a protease component of anthrax toxin as well as host proteases exploited by this toxin. We identified two approved drugs, ascorbic acid 6-palmitate and salmon sperm protamine, that effectively inhibited anthrax cytotoxic protease and demonstrated that they also block proteolytic activities of host furin, cathepsin B, and caspases that mediate toxin’s lethality in cells. We demonstrated that these drugs are broad-spectrum and reduce cellular sensitivity to other bacterial toxins that require the same host proteases. This approach should be generally applicable to the discovery of simultaneous pathogen and host-targeting inhibitors of many additional pathogenic agents.

The traditional method of treating most human diseases is to direct a therapy against targets in the host patient, whereas conventional therapies against infectious diseases are directed against the pathogen. Unfortunately, the efficacy of pathogen-oriented therapies and their ability to combat emerging threats such as genetically engineered and non-traditional pathogens and toxins have been limited by the occurrence of mutations that render pathogen targets resistant to countermeasures. Thus, host proteins exploited by pathogens are potential targets for therapies^1^.

Host proteins and pathways exploited by *Bacillus anthracis* toxins are well understood^2^. *B. anthracis* causes anthrax infections and leads to toxemia in humans and animals, rendering antibiotic therapies ineffective in the later stages of infection. The major virulence factors of the bacterium include an exotoxin protein complex consisting of protective antigen (PA) and lethal factor (LF), which act collectively to damage the host^2^. Proteases play important roles in anthrax toxin mediated host-cell killing. PA binds to host cellular receptors in the native form of 83 kDa (PA83)^3^,^4^, and once bound, host protease furin cleaves a 20 kDa fragment from the N-terminus of PA, thus activating the PA protein of 63 kDa (PA63)^5^. Following activation, PA forms a heptamer and binds LF^6^. The toxin undergoes clathrin-type endocytosis, mediated by another set of host proteases, calpains and cathepsin B^7^,^8^. A decrease in endosomal pH induces the formation of an endosomal membrane PA channel, by which LF translocates into the cytosol^9^.

Once in the cytosol, LF itself acts as a protease that cleaves and inactivates host mitogen-activated protein kinase kinases (MAPKK) 1–4, 6, and 7^10^. The MAPKK cleavage event prevents the passage of signals in the ERK1/2, p38, and c-Jun N-terminal kinase pathways^10^,^11^, which mediate responses to a variety of cellular stresses. In addition, rat NLRP1 and mouse NLRP1b proteins can also be directly cleaved by LF at sites near their N termini^11^,^12^. The cleavage of host proteins by LF results in the activation of the inflammasomes, resulting in rapid macrophage cell death mediated by additional host proteases, caspases-1 and -3^11^,^12^.

While the discovery of LF inhibitors has focused on new chemical compounds that either inhibit its protease activity or its cytoplasmic entry (reviewed in^13^), repurposing of existing drugs that simultaneously inhibit LF and the host proteases that assist LF, offers potential advantages. We used a fluorescence resonance energy transfer (FRET) assay, where LF cleaves a MAPKK2 peptide, to screen and identify approved drugs that affect the rate of the proteolytic reaction. We identified chemical and peptidic compounds that effectively inhibited cleavage of MAPKK2 peptide, as well as host furin, calpain, cathepsin B, and caspases. Two of those chemicals, ascorbic acid 6-palmitate and salmon sperm protamine, suppressed LF-induced cell death, as well as the cytotoxicity induced by cholera toxin and *Pseudomonas aeruginosa* exotoxin A. This study offers new solutions to treat these infectious diseases by using drugs that cross-inhibit pathogen and host targets.

## Results

### Observation of functional similarities between pathogenic agents and the host proteins exploited by them

Cytotoxic bacterial and plant toxins have evolved to exploit host proteins and cellular pathways that mediate the entry of those toxins into host cells and induce cell-death pathways. We observed a widespread phenomenon of structural or functional similarity between pathogenic proteins of bacteria, viruses, fungi, or other parasites and the host proteins that are exploited by them (Table 1). For example, similarities were reported for proteases of anthrax^7^,^8^,^14^,^15^ and botulinum toxins^16^,^17^, as well as HIV-1^18^,^19^,^20^,^21^ and Hepatitis C^22^,^23^,^24^ proteases and endocytosis-mediating host proteases. Furthermore, shiga glycosidase H toxin exploits host glycosidase H^25^; *Candida albicans* cell wall adhesins bind to structurally similar host cadherins during fungal invasion^26^; and Streptokinase and Staphylokinase exploit host plasminogen activators kinases^27^,^28^. A drug screen against multiple proteins within the same pathway is possible if these proteins are similar in function or structure. Therefore, finding therapies that cross-inhibit multiple proteins within a single pathway is of great interest.

**Figure 1:**
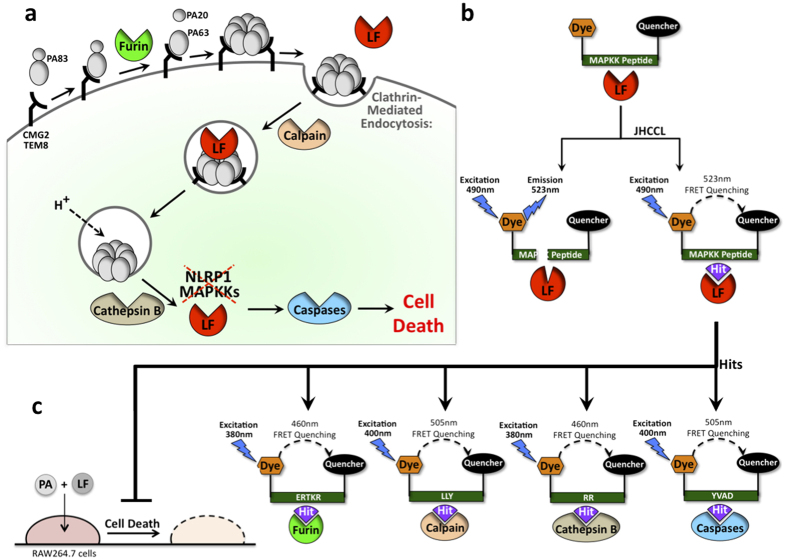
The use of the Johns Hopkins Clinical Compound Library (JHCCL) to screen for inhibitors of anthrax lethal toxin and host proteases. (**a**) Schematic depiction of host protease components within the pathway that mediates the delivery of anthrax toxin into cytoplasm. Lethal factor (LF) interact with a second *B. anthracis* protein, protective antigen (PA), whose role is to bind to host cell receptors, CMG2 and TEM8. Three host cell proteins furin, calpain, and cathepsin B mediate the endocytosis of the toxin complex. Once in the cytoplasm, LF cleaves host MAPKK’s and NLRP1, and initiates apoptosis through induction of proteolytic activity of host caspases-1 and -3. (**b,c**) Overall approach scheme: JHCCL is screened by biochemical FRET assay looking for drugs that are able to reduce proteolytic activities of anthrax toxin and five host proteases that mediate toxin lethality. (**b**) Schematic diagram of FRET screen to identify drugs that inhibit proteolytic reaction of LF, (**c**) followed by FRET reactions to test the ability of LF inhibitors to also inhibit proteolytic reactions mediated by host furin, calpain, cathepsin B, caspase-1, and caspase-3. In addition, LF FRET inhibitors are tested for their ability to reduce LF-PA cytotoxicity of mouse macrophage cells.

In an effort to identify existing drugs that might be repurposed as novel cross-inhibitors of pathogenic agents and the host proteins exploited by them, we screened the Johns Hopkins Clinical Compound Library (JHCCL) of chemicals^29^. We looked for compounds capable of i) inhibiting the proteolytic activities of *Bacillus anthracis* lethal toxin and the host proteases exploited by it (Fig. 1a) in biochemical FRET assays (Fig. 1d), and ii) reducing cytotoxicities of *B. anthracis* lethal toxin, Cholera toxin, and *Pseudomonas aeruginosa* exotoxin A (Fig. 1c). These toxins were chosen for this study because their pathways of entry into host cells are well understood^30^, making these toxins good tools for identification of broad-spectrum drugs.

### A biochemical screen for JHCCL drugs that cross-inhibit proteolytic activities of anthrax toxin and host proteases that mediate its lethality

In our initial screen we searched for the drugs which were capable of simultaneously inhibiting the proteolytic activities of anthrax LF as well as of host proteases that mediate the toxin’s lethality in cell-free FRET assays. To screen and identify drugs that inhibit LF proteolytic activity we utilized a fluorescence-based FRET assay. An optimized MAPKK2 peptide with a fluorogenic FITC group at the N terminus and DABCYL quenching group at the C terminus was used as the LF substrate. After cleavage by LF the fluorescence of FITC at 523 nm increases, while a known inhibitor of LF, surfen hydrate^31^, prevents it from cleaving the substrate and producing fluorescence (Fig. 1b). We screened 1,585 previously approved drugs for their ability to reduce the rate of the LF proteolytic reaction. Surfen hydrate is not included in JHCCL because is it not used clinically. Compounds that showed greater than 66% (~1% hit rate) inhibition when tested at 33 μM were selected for re-validation and further studies. We discovered ten compounds that effectively inhibited LF in the FRET assay, nine of which were chemical molecules and one which was a 33 amino acid long peptide called salmon sperm protamine (Table 2). Of the ten chemical hits, six drugs were structurally similar to each other and contained carbon chains of at least 12 carbons (Table 2). These drugs are cetylpyridinium bromide, domiphen bromide, cetalkonium chloride, sodium lauryl sulfate, ascorbic acid 6-palmitate, and sodium dodecylbenzenesulfonate. Three other small molecule drugs, docusate sodium, thyropropic acid, candesartan cilexetil, successfully inhibited the LF proteolytic reaction and were of various structures (Table 2). Moreover, one of the drugs that successfully inhibited the LF FRET reaction was a peptide, salmon sperm protamine. We determined that the IC50 values for the LF FRET reaction of these ten drugs were in the high nM to low μM range (Table 2), with cetalkonium chloride showing the lowest IC50 of 160 nM, while arginine-rich salmon protamine displaying the lowest potency of 18 μM.

We tested several truncated versions of salmon protamine and showed that while the N’ terminal region of the peptide (amino acids 1–10) lost the anti-LF and anti-furin FRET efficacies, and the C’ terminal (amino acids 23–33) showed potency similar to the full length protamine, the central portion of protamine (amino acids 11–22) displayed improved anti-LF activity (Table 3). Additionally we observed that a truncated version of protamine that contains the N’ terminal and central portions of protamine showed an even further improvement of protamine anti-toxin efficacy of just 1 μM. A truncated version of protamine that contains the central and C’ terminal regions of protamine demonstrated anti-LF efficacy that is comparable to the full length protamine.

We observed that arginine-rich human sperm protamine exhibited an anti-LF FRET IC50 comparable to that of salmon protamine (Tables 3 and 4). We tested several truncated versions of human protamine and showed that while the N’ terminal region of the peptide (amino acids 1–17) showed a decreased anti-LF FRET efficacy, central (amino acids 18–34) and C’ terminal (amino acids 35–51) regions showed potency similar to the full length human protamine (Table 4).

All ten LF-inhibitors were also tested for their ability to inhibit proteolytic FRET reactions of host furin, calpain, cathepsin B, caspase-1, and caspase-3, all of which mediate LF cytotoxicity (Fig. 1c). With the exception of caspase-1, all human proteases were effectively inhibited by several LF inhibitors (Table 2). The only drug that exclusively inhibited LF was domiphen bromide. None of the drugs inhibited all proteases.

### Ascorbic acid 6-palmitate and salmon sperm protamine inhibit cytotoxic activity of anthrax toxins

**Figure 2:**
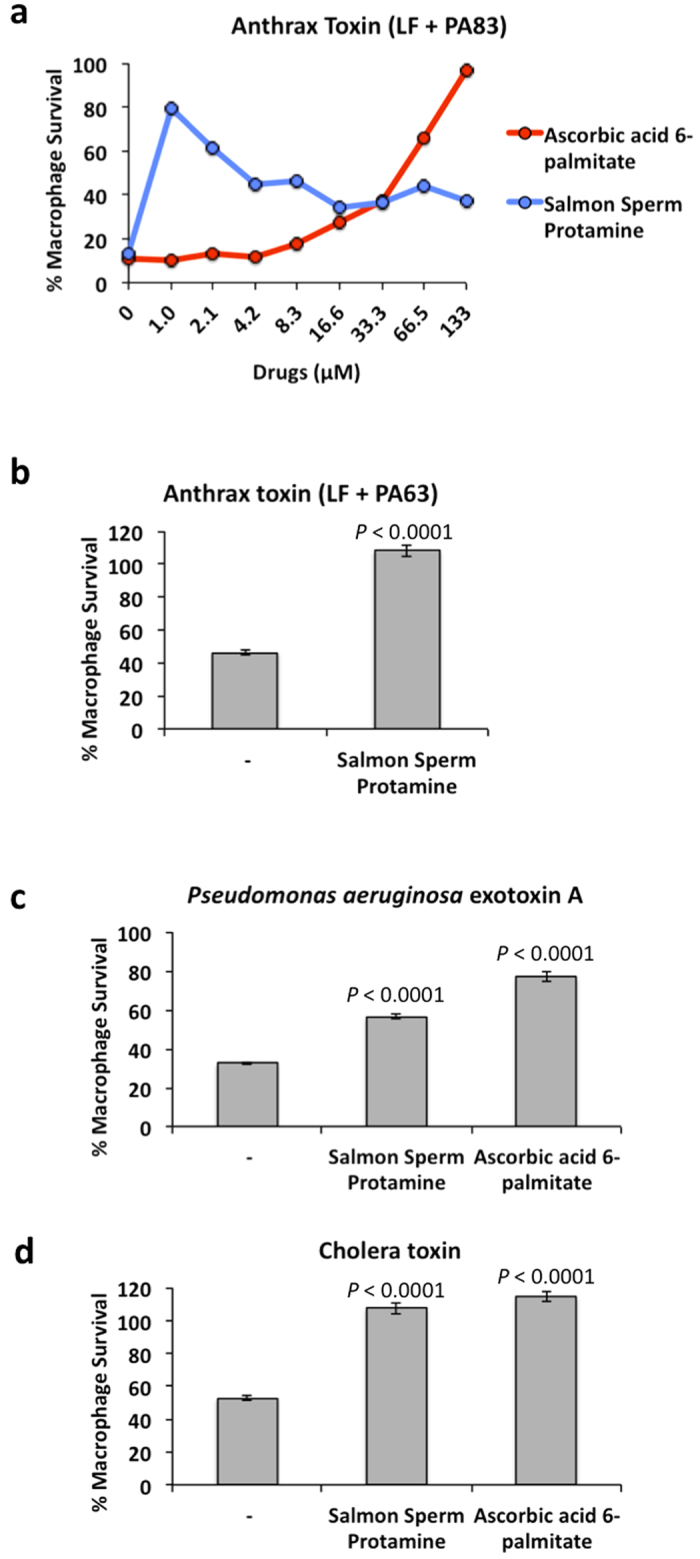
Ascorbic acid 6-palmitate and salmon sperm protamine act as broad-spectrum anti-toxins. (**a**) Ascorbic acid 6-palmitate and salmon sperm protamine were tested for their ability to inhibit LF-PA83-mediated cytotoxicity. RAW264.7 cells were seeded at 1 × 10^4^ cells/well on 96-well plates and the following day were incubated with indicated doses of drugs for 1 hour, followed by 3 hours intoxication with 0.5 μg/ml PA83 + LF. (**b**) Sixteen μM of salmon sperm protamine reduces cellular sensitivity to LF + PA63. RAW264.7 cells were pretreated either with DMSO or with protamine for 1 hour, and then treated with 0.5 μg/ml of LF and PA63 for 6 hours. Averages, standard deviations, and *P* values are shown for each condition. (**c,d**) Ascorbic acid 6-palmitate and salmon sperm protamine were tested for their ability to inhibit cytotoxicities mediated by *Pseudomonas aeruginosa* exotoxin A and Cholera toxin. RAW264.7 cells were seeded at 1 × 10^4^ cells/well on 96-well plates and the following day were incubated with indicated doses of drugs for 1 hour, followed by 12 hours intoxication with 2 and 4 μg/ml of *Pseudomonas* (**c**) and Cholera (**d**) toxins respectively. Cell viability was determined by MTT assay (Materials and Methods) and is shown as the percentage of survivors relative to cells not treated with drugs. Averages, standard deviations, and *P* values are shown for each condition. Each *P* value represents a comparison of drug-treatment condition to a condition without drugs.

In order to test the ability of LF inhibitors to neutralize the cytotoxic activity of anthrax toxin (Fig. 1c), we examined the effect of ten JHCCL hits on cell viability in LF-PA treated mouse macrophage RAW264.7 cells. Between 80 and 90 percent of cells used for these assays normally undergo cell death within 3 hours of exposure to anthrax lethal toxin under the experimental conditions employed. Ascorbic acid 6-palmitate and salmon sperm protamine were the only two drugs that provided complete protection against LF-PA mediated cell killing (Fig. 2a). In contrast, the remaining eight compounds failed to protect cells against LF-PA mediated cellular killing due to their cytotoxicity in RAW264.7 cells. These experiments indicate that ascorbic acid 6-palmitate and salmon sperm protamine display target-specific biological activity and are therefore candidates for further development into broad-spectrum therapeutics.

Poly-arginine peptides were previously shown to simultaneously inhibit furin and lethal factor in biochemical and cellular assays^32^,^33^. We tested the ability of arginine-rich protamine to reduce the cell death induced by LF and PA in the 63 kDa form - lacking the 20 kDa furin cleavage domain (Fig. 2b). Fifty percent of cells used for these assays normally undergo cell death within 6 hours of exposure to LF-PA63 under the experimental conditions employed. We observed that protamine effectively protected all cells when used at 16 μM, compared to cells treated with LF-PA63 in the absence of drugs (*P* < 0.0001). Since this form of PA bypasses the need for furin protease cleavage, this data argues that protamine reduces toxin-mediated cell death by inhibiting LF directly.

### Ascorbic acid 6-palmitate and salmon sperm protamine act as broad-spectrum anti-toxins

It has been previously shown that *Pseudomonas aeruginosa* exotoxin A (PE) and Cholera toxin exploit several host proteins for their binding and entry into host cells and initiate programmed cell death by inducing activities of host caspase-3^34^,^35^. To test the ability of ascorbic acid 6-palmitate and salmon sperm protamine to neutralize cytotoxic activity of the two toxins, we examined their effects on cell viability in toxin-treated RAW264.7 cells. While 50% and 70% of cells used for these assays normally undergo cell death within 6 hours of exposure to CT and PE respectively, ascorbic acid 6-palmitate and salmon sperm protamine provided substantial protection against PE and CT-mediated cell killing at 16 μM compared to cells treated with toxins in the absence of drugs (*P* < 0.0001) (Fig. 2c,d).

## Discussion

This study identified drugs that simultaneously target pathogenic factors and host proteins exploited by them. This approach is based on the observation that some virulence proteins and host proteins they utilize, belong to the same functional class. However, this approach has possible limitations such as potentially low efficacy of drugs, or if a pathogenic factor exploits multiple redundant host proteins, some of which could be functionally unrelated to the toxin. Moreover, host protein inhibiting therapies may cause side effects, and this is a possible explanation as to why many drugs that were shown to have potency in biochemical FRET assays were cytotoxic in cellular anti-toxin assays (Fig. 2). Some of the drugs that caused the cytotoxicity display structural properties that resemble detergents, while other drugs could be cross-reacting with essential host proteins.

Detergents are compounds comprised of a hydrophobic hydrocarbon tail and a hydrophilic charged headgroup. When dissolved in water at a given concentration, detergent molecules will form micelles, with the hydrophobic tail in the interior of the micelle and the headgroup at the exterior. The minimal detergent concentration at which micelles are observed at a specific temperature is called “Critical Micelle Concentration” (CMC). It is known that at or above the CMC, detergents cooperatively bind to most proteins, which thereafter undergo denaturing rearrangements of their protein structures. Micelles act as detergents with the hydrophobic core of the micelle binding to the hydrophobic regions of proteins. It is also known that at submicellar concentrations, detergents may bind to specific binding sites of several proteins^36^,^37^. For example, the CMC of one of our FRET hits, sodium lauryl sulfate, a molecule commonly known as SDS, is 8 mM^38^. In our study the IC50 of SDS was found to be 2.9 μM, four orders of magnitude of concentrations below the CMC. Similarly, the IC50 of ascorbic acid 6-palmitate in our study is 0.4 μM, while its CMC is 0.8 mM^39^, four orders of magnitude difference. Therefore, since all of our detergent-like molecules displayed potencies well below their respective CMC’s, we believe that their interaction with the studied protein targets is specific. The data shown in Table 2 argues that these drugs are selective in their inhibition of targets, and none of the drugs inhibited all targets indiscriminately. Lastly, with the exception of ascorbic acid 6-palmitate, all detergent-like molecules discovered in this study belong to the common ionic category of detergents, as their headgroups bear a charge. In contrast, ascorbic acid 6-palmitate is a non-ionic molecule, and this class of detergent-like molecules are known to less likely act as detergents.

Protamines are arginine-rich peptides that package DNA into chromatin in vertebrate sperm. It is thought that the N’ terminus of protamine is unstructured, contains a lesser number of arginine residues, and bends back and over the central arginine-rich portion of protamine before binding the DNA^40^. There could be several reasons why the N’ termini of salmon and human protamines are less potent than their full-length equivalents. The N’ termini of protamines contain the lowest number of arginines, which could be necessary for inhibiting LF protease activity. We hypothesize that in addition to arginine numbers, the secondary structure of protamine is also important, as protamine constructs that include the N’ terminal and central portions are the most potent anti-LF peptides.

The anti-toxin efficacies of drugs in cellular assays were observed to be lower than the efficacies seen in FRET assays, possibly because some of the host protein targets are intracellular, and drugs are required to cross cell membrane barrier. Moreover, the experimental timing used to determine drugs’ inhibitory activity on proteases in FRET assays is very different from the timing used to measure the ability of drugs to block toxins in cellular assays. Cellular assays are done by pre-incubating cells with drugs for 1 hour, following by 6–24 hours of toxin treatments, depending on the kind toxin used. In contrast, FRET reactions were performed for up to 2 hours without drug pre-incubation. Finally, one protease activity unit of a single protease was used per FRET reaction. In contrast, the number of protease activity units per one cell is not known and may not be the same used in FRET assays.

The pharmacokinetics and safety of both identified drugs are well characterized. Ascorbic acid 6-palmitate is fat-soluble form of vitamin C formed from ascorbic acid and palmitic acid. It is used as a source of vitamin C as well as an antioxidant food additive (E number E304), which is approved for use as a food additive in the EU, the U.S., Australia, and New Zealand^41^. Salmon sperm protamine is used as an antidote for heparin overdoses^42^. In addition, protamine is used to slow down the onset and increase the duration of insulin action^43^.

Numerous screens for LF inhibitors have been attempted in the past decade^13^, however, none of the identified hits have been approved for use in humans because of the long and rigorous process of drug discovery and development. The current drug discovery and development process can take 8 to 12 years for the successful introduction of a new drug into the market^44^. Thus, the current *de novo* drug discovery and development paradigm is ineffective for dealing with rapidly emerging biological threat agents. Drug repurposing may offer numerous advantages under these circumstances. Since currently approved drugs already have well-established safety and pharmacokinetic profiles in patients as well as bulk manufacturing and distribution networks, they could rapidly be made available for a new indication if a biological emergency were to occur.

## Experimental Procedures

### Chemicals and Reagents

All bacterial toxins were purchased from List Biological Laboratories (Campbell, CA, USA). An FDA-approved drug library comprising of 1,585 drugs was purchased from Johns Hopkins University Bloomberg School of Public Health, titled, Johns Hopkins Clinical Compound Library (JHCCL). The drugs in the library were kept at −20 °C as 3.3 mM stock solutions in sealed microtiter plates and were made using DMSO as solvent. Compounds of interest were repurchased and reproduced from 3.3 mM solutions. Salmon sperm protamine, cetylpyridinium bromide, domiphen bromide, cetalkonium chloride, sodium lauryl sulfate, ascorbic acid 6-palmitate, sodium dodecylbenzenesulfonate, docusate sodium, and candesartan cilexetil were purchased from Sigma-Aldrich (St. Louis, MO, USA). Thyropropic acid was purchased from Toronto Research Chemicals Inc (Toronto, Ontario, Canada). All drugs were prepared at 3.3 mM using DMSO as a solvent. Full-length human and truncated salmon and human protamines were synthesized by LifeTein (Hillsborough, NJ, USA). All chemicals and peptides in this study had purities greater than 98%.

### LF FRET Drug Screen and Data Analysis

For screening JHCCL drugs in 96-well plates, the reaction volume was 250 μl per well, containing 20 mM HEPES pH 7.2, 5 μM MAPKKide conjugated with DABCYL and FITC (List Biological Laboratories, Inc), and 33 μM of JHCCL compound. Reactions were initiated by adding anthrax LF to a final concentration of 6 μg/ml. Kinetic measurements were obtained at 37 °C every 40 sec for 50 min using a fluorescent plate reader. Excitation and emission wavelengths were 490 nm and 523 nm, respectively, with a cutoff wavelength of 495 nm. A known LF inhibitor, surfen hydrate^31^, was included as a control. Rates of reactions were quantified by the Microsoft Excel LINEST function. Drugs that inhibited at least 66% of FRET reaction at 33 μM were re-tested at decreasing concentrations in order to determine IC50.

### Host Proteases FRET Tests

Drug hits that inhibited at least 66% of LF FRET reaction were also tested for their ability to inhibit FRET reactions of human furin (New England Biolabs), calpain 1 (BioVision), cathepsin B (EMD Millipore), and caspases-1 and -3 (BioVision). The fluorescently labelled substrate peptide for furin FRET assay was purchased from Peptides International. The positive control furin Inhibitor I was purchased from EMD Millipore. The calpain Inhibitor 1, ALLN, was purchased from BioVision and used as a control. Amodiaquine (Sigma-Aldrich) was used as a control inhibitor of cathepsin B. The universal caspase inhibitor Z-VAD-FMK (BioVision) was used as a control. Rates of reactions were quantified by the Microsoft Excel LINEST function.

### Cell Culture, Toxins Treatments, and Cell Viability Assays

RAW264.7 mouse macrophage cells were maintained in DMEM (Invitrogen) supplemented with 10% FBS (Corning) and 100 μg/ml penicillin and streptomycin. RAW264.7 cells (10,000 per well) were seeded in 96-well plates (100 μl/well) 24 hours before the assay. Cells were treated with drug hits for 1 hour at 37 °C 5% CO_2_. RAW264.7 cells were challenged with anthrax toxins that include LF and native 83 kDa PA (for 3 hours), LF and PA in the 63 kDa form (for 6 hours), *P. aeruginosa* exotoxin A (for 12 hours), or Cholera toxin (for 12 hours), which were also pre-treated with identical drug concentrations, such that the final toxins concentrations were 0.5, 0.5, 2.0, and 4.0 μg/ml respectively. Determination of RAW264.7 viability was performed by 3-(4,5-dimethylthiazol-2-yl)-2,5-diphenyltetrazolium bromide (MTT) assay was performed as described^1^. Cell viability is defined as the percentage of surviving cells obtained relative to cells treated with DMSO (100%). At least three such experiments were carried out. Statistical analysis was performed using GraphPad Prism software (http://www.graphpad.com/scientific-software/prism/). Each *P* value represents a comparison of drug-treatment condition to a condition without drugs, and values < 0.05 were considered statistically significant.

## Additional Information

**How to cite this article**: Zilbermintz, L. *et al*. Cross-inhibition of pathogenic agents and the host proteins they exploit. *Sci. Rep.* **6**, 34846; doi: 10.1038/srep34846 (2016).

## Figures and Tables

**Table 1:** The observation that many pathogenic and host proteins they exploit belong to the same functional class.

Pathogenic agent	Exploited host protein	Shared function	References
Anthrax Lethal Toxin	Furin, calpain, cathepsin B, caspase-1, caspase-3	Proteases	7,8,14,15
*Botulinum* neurotoxins	caspase-3/7	Proteases	16,17
*Candida albicans* Als3	E-cadherin and N-cadherin	Adhesins	26
Hepatitis C NS2-3 and NS3-4	Furin, calpain, caspase-3	Proteases	22, 23, 24
HIV-1 PR	Furin, calpain, cathepsin B, caspase-1/3	Proteases	18, 19, 20, 21
*Pasteurella Multocida* toxin	A2B Adenosine Receptor	Adenosine deaminases	45
*Plasmodium berghei* ALAS and FR	ALAS-1 and FECH	Heme-biosynthesis	46
*Shiga* toxin	Endoglycosidase H	Glycosidases	25
Staphylokinase and Streptokinase	Plasminogen activators (PLAT and PLAU)	Kinases	27,28

**Table 2:**
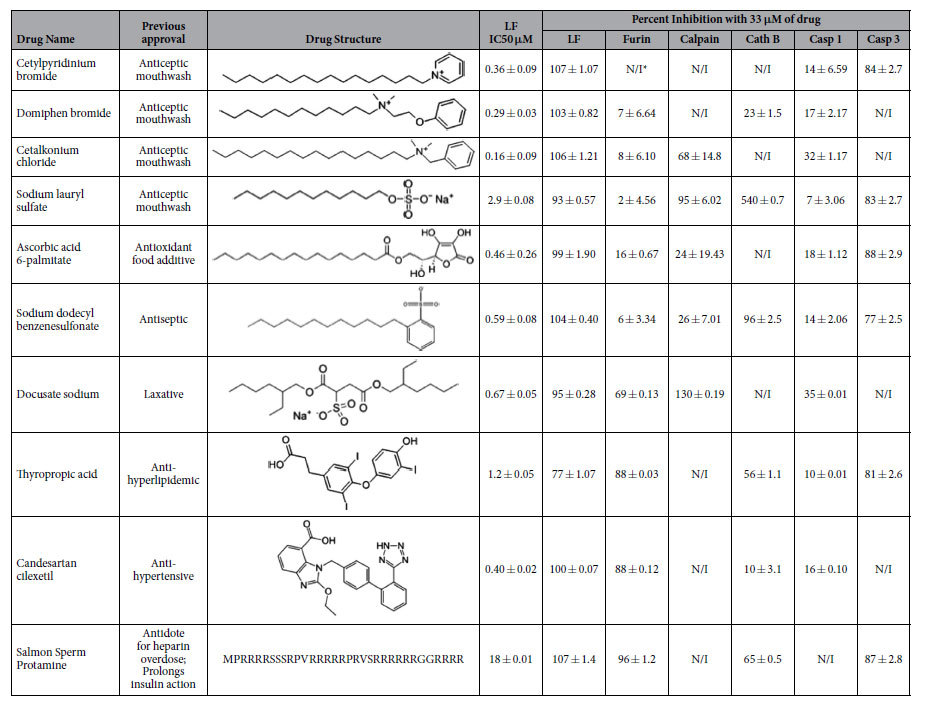
The potencies of ten JHCCL drugs for inhibition of anthrax LF, as well as human proteases exploited by it (furin, caspase, cathepsin B (Cath B), caspase-1 (Casp 1), and caspase-3 (Casp 3)) in biochemical FRET assays.

Conditions where no FRET inhibition is observed denoted as N/I.

**Table 3:** The potencies of truncated versions of salmon sperm protamine and full-length human protamines in LF and furin FRET assays are shown. N/I stands for no inhibition.

Peptide Name (amino acids)	Peptides amino acid sequences	LF IC50 μM	Percent Inhibition with 33 μM of drug	Furin
			LF	
Salmon Sperm Protamine (1-33)	MPRRRRSSSRPVRRRRRPRVSRRRRRRGGRRRR	18 ± 0.01	107 ± 1.4	96 ± 1.2
Salmon Sperm Protamine (1-10)	MPRRRRSSSR	298 ± 0.01	28 ± 1.0	N/I
Salmon Sperm Protamine (11-22)	PVRRRRRPRVSR	5 ± 0.13	95 ± 0.1	84 ± 33
Salmon Sperm Protamine (23-33)	RRRRRGGRRRR	15 ± 0.01	93 ± 2.4	150 ± 5.3
Salmon Sperm Protamine (1-22)	MPRRRRSSSRPVRRRRRPRVSR	1 ± 0.04	98 ± 0.6	91 ± 1.8
Salmon Sperm Protamine (11-33)	PVRRRRRPRVSRRRRRRGGRRRR	10 ± 0.04	99 ± 0.2	86 ± 1.2
Human Sperm Protamine (1-51)	MARYRCCRSQSRSRYYRQRQRSRRRRRRSCQTRRRAMRCCRPRYRPRCRRH	14 ± 0.32	88 ± 1.2	97 ± 1.7

**Table 4:** The potency of full length and truncated versions of human protamine in LF FRET assay.

Peptide Name (amino acids)	Peptides amino acid sequences	LF IC50 μM	Percent Inhibition with 33 μM of drug
			LF
Human Sperm Protamine (1-51)	MARYRCCRSQSRSRYYRQRQRSRRRRRRSCQTRRRAMRCCRPRYRPRCRRH	14 ± 0.32	88 ± 1.2
Human Sperm Protamine (1-17)	MARYRCCRSQSRSRYYR	67 ± 0.01	37 ± 0.7
Human Sperm Protamine (18-34)	QRQRSRRRRRRSCQTRR	24 ± 0.15	72 ± 2.1
Human Sperm Protamine (35-51)	RAMRCCRPRYRPRCRRH	28 ± 0.03	55 ± 0.9
